# Incidence and Pattern of Dog Bite Injuries Treated in the Emergency Room of a Teaching Hospital South East Nigeria

**Published:** 2018

**Authors:** Njoku Isaac Omoke, Ndubuisi Onu Chukwueloka Onyemaechi

**Affiliations:** 1 Department of Surgery, Ebonyi State University, Federal Teaching Hospital, Abakaliki; 2 Department of Surgery, College of Medicine, University of Nigeria, Ituku/Ozalla, Enugu, Nigeria

**Keywords:** Dog bite, emergency room, injury, Nigeria, rabies

## Abstract

**Background:**

Dog bite injury treated in the emergency room varies from and within subregions in pattern and potential risk of transmission of rabies. This variation has implications in its morbidity and mortality. The aim of this study was to determine the incidence and pattern of dog bite injuries treated in a teaching hospital emergency room setting of a developing country.

**Patients and Methods:**

This was a retrospective study of the entire patients with dog bite injury treated in the emergency room of Federal Teaching Hospital Abakaliki from January 2006 to December 2015.

**Results:**

Dog bite injury necessitated visit in 74 patients with an incidence of 2 per 1000 emergency room attendances, and a male to female ratio of 1:1.1. The mean age of the patients was 25.5 ± 1.87 years, and peak age group incidence was 5–9 years. Lower extremity was involved in 77.5% of the injuries, and buttock was the predominant site of injury in 0–4 years old. Fifty-one (68.9%) owned dogs and 23 (31.1%) stray dogs were involved in the attack. There was unprovoked attack in 81.1% of cases, and 51 (68.9%) sustained Grade II injury. Twenty-eight (37.8%) of the dogs had anti-rabies vaccination. Fifty-four (73%) patients had no prehospital care while 64 (86.5%) received postexposure anti-rabies vaccine. Majority of the patients 73 (98.7%) recovered fully. One (1.4%) patient that presented with clinical rabies self-discharged against medical advice.

**Conclusion:**

The incidence of dog bite injury is within worldwide range though the female gender bias is unprecedented. We recommend preventive strategies based on the observed pattern and improvement in the rate of prehospital care and higher coverage of anti-rabies vaccination of dogs.

## Introduction

*D*og bite injury in human is an important global public health problem, although underreported in the developing countries. In the USA, a survey conducted in 2001–2003 indicates that victims of dog bite are about 4.5 million each year, and 19% of them sustained injury necessitating medical attention.^[1]^ In published reports, the incidence of dog bite injury in high-income countries ranges from 0.73 to 22/1000 per annum,^[1–4]^ whereas the incidence in low- and middle-income countries ranges from 1.03 to 25.7/1000 per annum.^[5,6]^ In Northern Nigeria, a recently published report indicates a rising incidence of dog bite injuries among the general population.^[7]^ These reports of bite-related injuries associated with the dog, a domestic animal generally considered a good friend of man, give serious cause for concern.

Human-dog association came into existence several centuries ago, and there has been a remarkable adaptation of dogs to domestic life over these periods.^[8]^ Consequently, humankind has harnessed the resourceful potentials of dogs to meet social, game hunting, security, and healthcare-related needs.^[8,9]^ Despite adaptations of dog to human needs, its wild instinct including behavior that often times lead to humans attack remains intact and is a major setback in human-dog relationship. Thus, dog bites in human results in open soft-tissue injury and fractures of varying degrees of severity.^[10]^ The soft-tissue injury varies from scratches/punctures to avulsion and crush injuries that can be severe enough to necessitate reconstructive procedures.^[10]^ A dog bite can also result in traumatic amputation as well as other limb or life-threatening injuries.^[11]^ Local and systemic wound infections, disfigurement, posttraumatic stress disorder, and other wound healing complications such as hypertrophic scar and keloid are all components of short- and long-term morbidity associated with dog bite injury in humans.^[4,12,13]^ The potential risk of transmission of rabies is even a more serious but preventable burden associated with dog bites. However, in most developing countries, dog is the most important vector in transmission of rabies;^[5,7,14]^ and this has been attributed to poor compliance to anti-rabies vaccination of dogs and dog management practices that aids dog and human exposure to rabies such as extensive free roaming system in a poor environmental hygiene setting.^[9,15]^

The victims of dog bite often present in Emergency Department (ED) for medical attention. Thus, the reported incidence of ED-treated dog bite injuries ranges from 0.2% to 1.1% of all ED visits,^[16–18]^ and 0.8%–5.6% of all injury-related ED visits.^[16,19]^ Dog bite injuries received in the hospital emergency room vary from and within subregions with respect to population characteristics, dog, and injury characteristics as well as potential risk for transmission of rabies. Knowledge of incidence and pattern of dog bite injuries received in hospital emergency room in a setting can facilitate preventive strategies and measures aimed at achieving optimum care of victims. Limited data on dog bite injuries managed in the emergency room of Teaching Hospitals in West African subregion necessitated this study.

The aim of this study was to determine the incidence and pattern of presentation of dog bite injuries treated in the emergency room of a Teaching Hospital in a developing country.

## Patients and Methods

This was a retrospective study of all patients with dog bite injury treated in the Accident and ED of Federal Teaching Hospital Abakaliki between January 2006 and December 2015. The case notes of these patients were the source of data. Information such as demographic data of patients, the date, season and time of injury, location and setting of injury, grade and type of injury, anatomical site of injury, interval between injury and presentation to the hospital, prehospital care, comorbidities, associated injuries treatment, length of hospital stay, outcome, and complications was extracted from the patient case notes.

The time of injury was categorized into four 12–5.59 am, 6–11.59 am, 12–5.59 pm, and 6–11.59 pm. In the setting of this study, nighttime refers to 6–11.59 pm and 12–5.59 am whereas daytime refers to 6–11.59 am and 12–5.59 pm. The months from November to April are the dry season period whereas the months from May to October are the wet/rainy season. The severity of injury was graded as follows: Grade 0 = no apparent injury seen, Grade 1 = skin scratch with no bleeding, Grade 2 = minor wound with some bleeding, and Grade 3 = deep, multiple injuries or any wound requiring suturing.^[20]^ Information about the characteristics of dog such as ownership and anti-rabies vaccination status and fate of the dog was also extracted from the case notes.

Data analysis was carried out using Statistical Package for Social Sciences (SPSS) version 16.0 (SPSS Inc., Chicago IL, USA). Frequency tables, cross tabulation, Fisher’s exact test, and Pearson’s Chi-square test of significance were used. For all statistical analysis *P* < 0.05 was considered statistically significant.

## Results

Within the 10 years’ period, 35,748 patients (19,862 males and 15,886 females) were treated in the hospital ED, and dog bite injury was the reason for visit in 74 (36 males and 38 females) of them, giving an incidence 2 per 1000 of ED attendance (1.8/1000 male and 2.3/1000 female ED attendance), and male to female ratio of 1:1.1. Patient’s age ranged from 2 to 62 years with a mean of 25.1 ± 1.87 years. Overall, the peak age group of the victims was 5–9 years.

**Figure 1:**
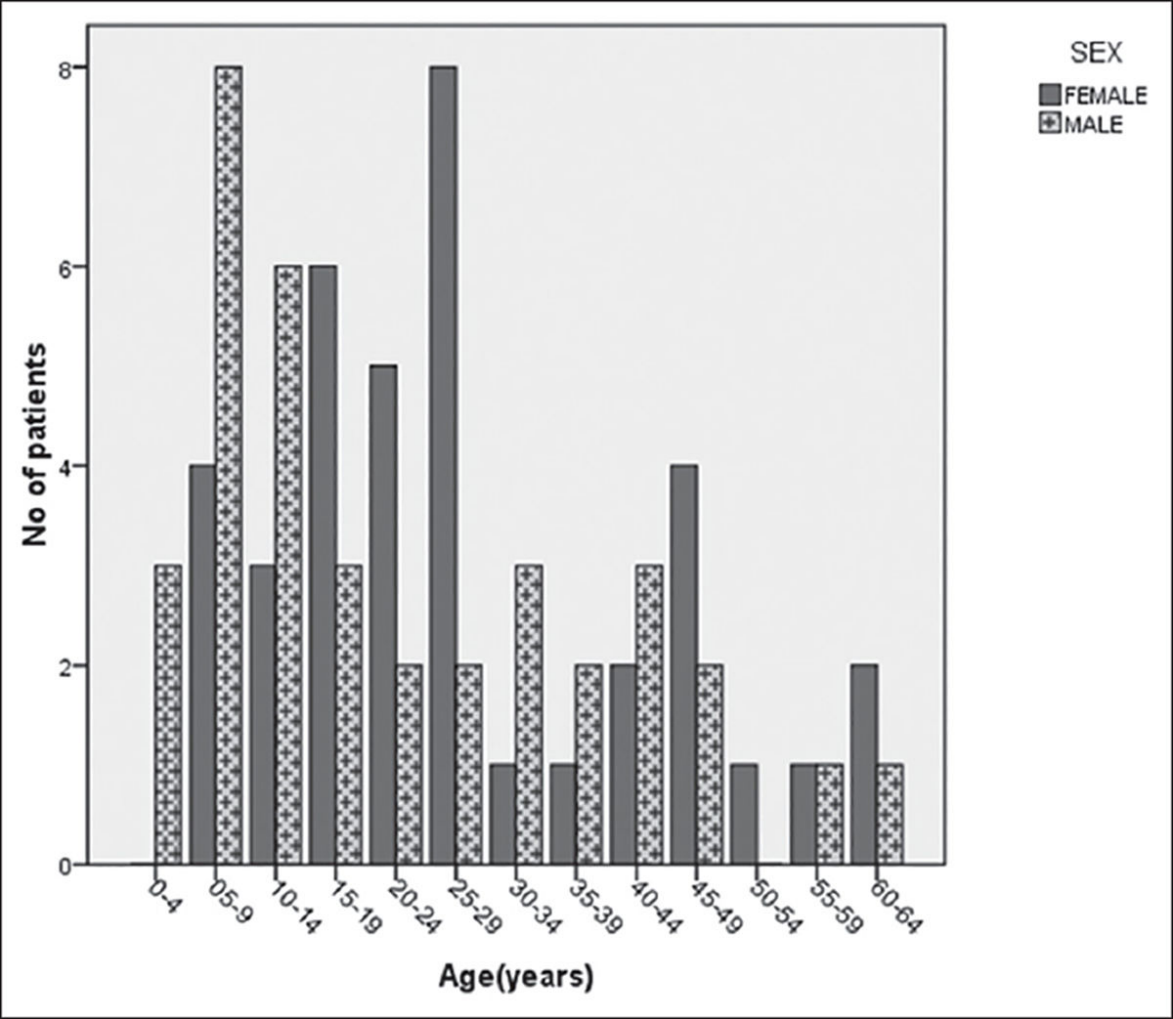
Distribution of dog bite injuries by age and gender

There was variation in the age distribution of victims by gender, among the males, after the first 4 years of life, dog bite injury quadrupled to a peak age incidence of 5–9 years whereas among the females the peak age incidence was 25–29 years, as shown in Figure 1. The preponderance of males in the first 14 years of age reversed to female preponderance in the age groups within 15–29 years.

**Figure 2:**
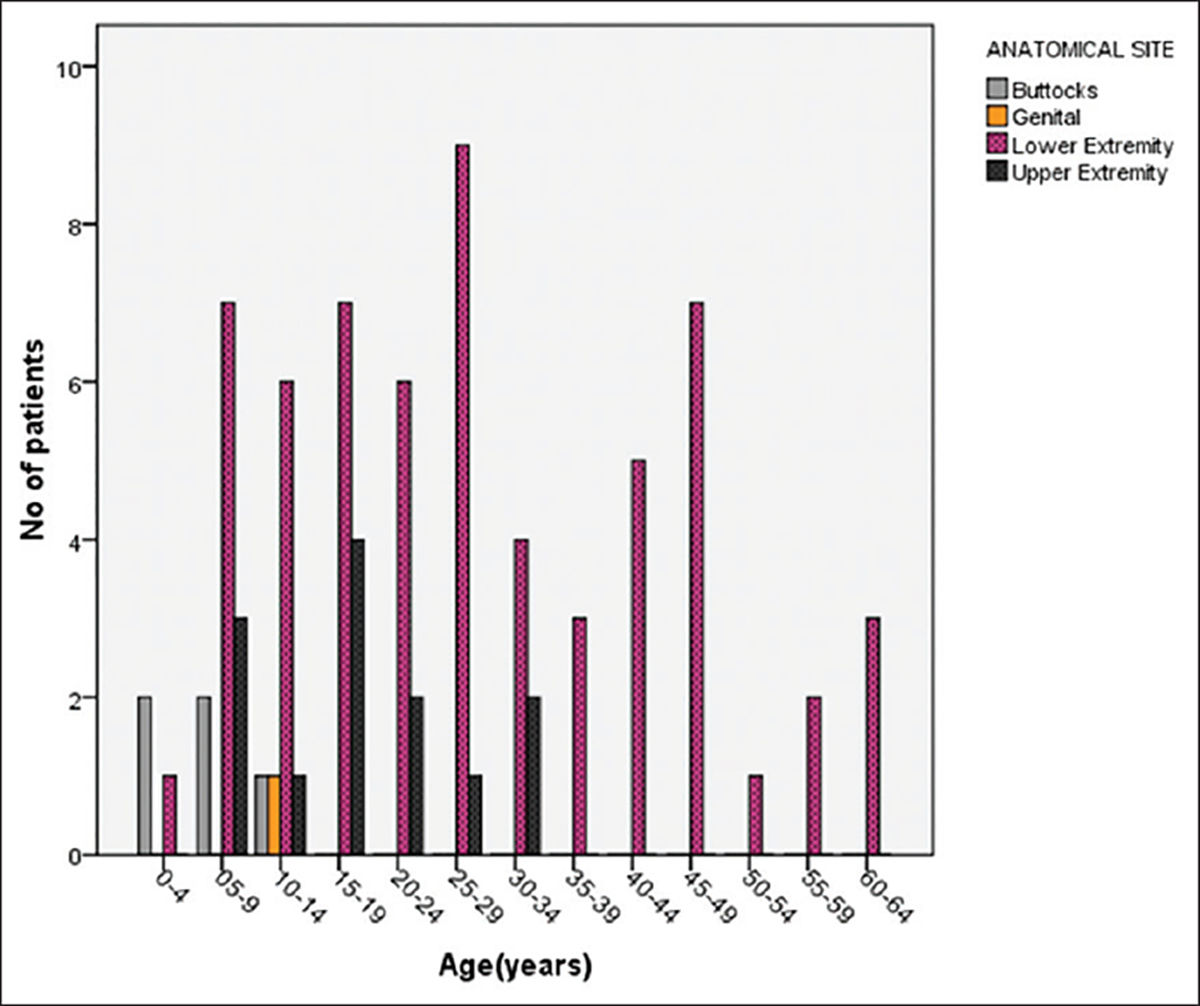
Distribution of dog bite injuries by age and anatomical site

The age distribution of victims by anatomical site of injury also varies. In Figure 2, from 5 to 9 years of age group onward, the incidence of lower extremity injury increased to a peak at the age of 25–29 years. The incidence of upper extremity injury increased to a peak in 5–9 years of age group then decreased with increasing age. The incidence for injury involving the buttocks was at its peak in the 0–4 and 5–9 years of age group, decrease by half in the 10–14 years of age group then rare afterward.

**Figure 3:**
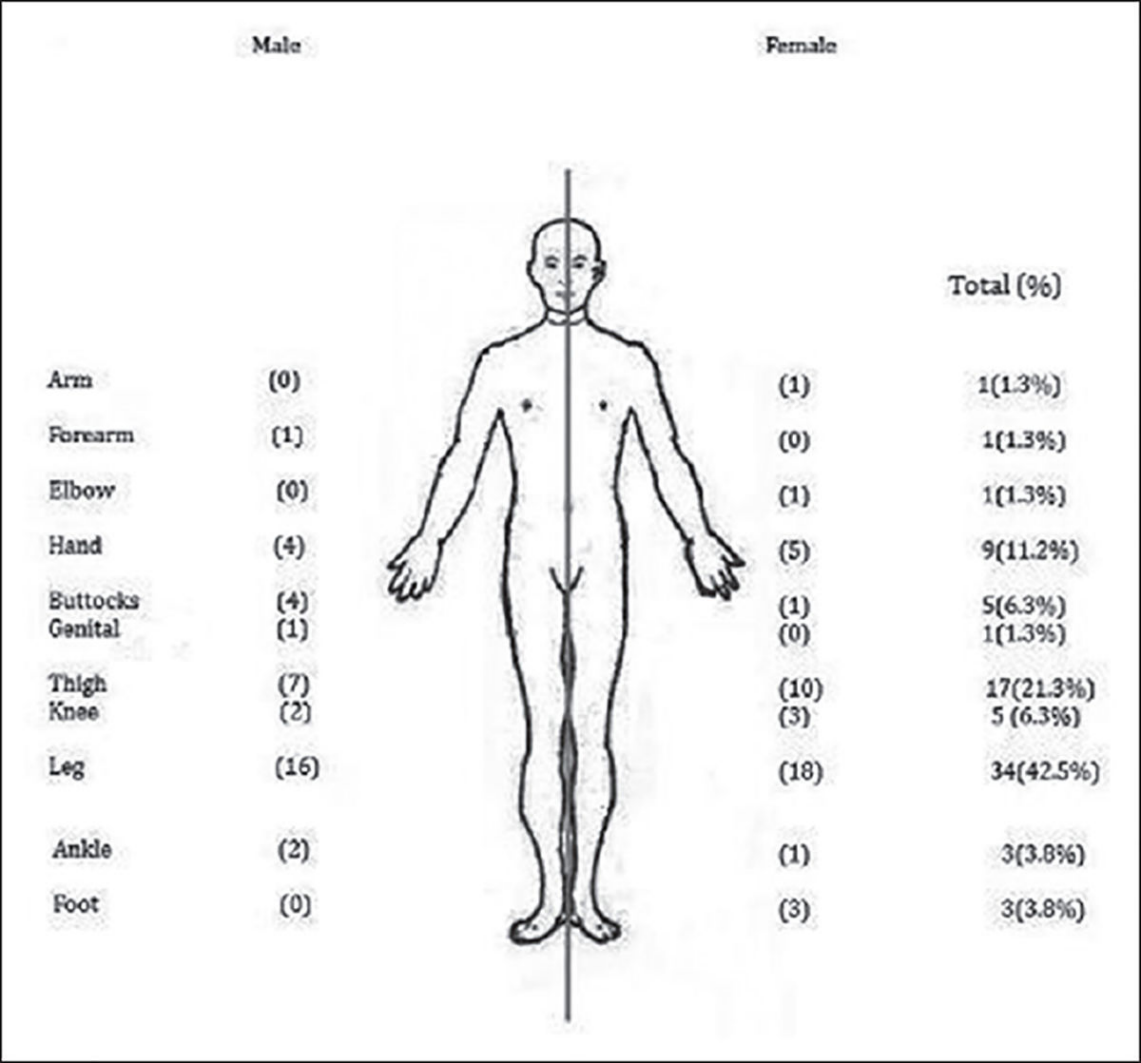
Distribution of dog bite injuries by gender and anatomical site

Dog bite injuries in lower and upper extremities respectively accounted for 62 (77.5%) and 12 (15%) of the 80 injuries observed in the 74 victims. In Figure 3, the leg (42.5%), thigh (21.3%), and hand (11.2%) were the three top anatomical sites involved in injury. The female victims sustained more injuries in the lower extremity than the males (35 vs. 27 injuries) whereas the later had more injuries in the buttocks than the former (4 vs. 1 injury). Injury to the genitalia (penis) accounted for 1.3% of all the injuries, and the victim was a 14-year-old boy.

There were 51 (68.9%) owned dogs and 23 (31.1%) stray dogs involved in the attack. The circumstances of bite were unprovoked in majority (81.1%) of the victims, and 51 (68.9%) sustained Grade II injury. All the victims sustained only soft-tissue injuries, scratches in 4 (5.4%), puncture wounds in 55 (74.3%), and lacerations in 15 (20.3%) of the patients, as shown in Table 1. Four (6.7%) and three (5%) of the 60 dogs involved in unprovoked bites had a history of previous bites and illness respectively at the time of the attack. Among the 15 lactating dogs involved in attack, the circumstance of bite was provoked and unprovoked in 3 (20%) and 12 (80%) of them, respectively.

Majority of dog bites, 59 (79.7%) occurred in the daytime (6–5.59 pm) whereas 15 (20.3%) occurred at nighttime (6–5.59 am). There was preponderance of dog attack in the urban areas, and majority of victims were bitten in their own/neighbors’ compound. Dog attack occurred at homes and compounds in 24 (63.2%) of the females and 24 (66.7%) of the males whereas 14 (36.8%) of the females and 9 (25%) of males were attacked in the street/major roads, as shown in Table 2.

There was history and claims of anti-rabies vaccination in 28 (37.8%) of the dogs involved whereas 23 (31.1%) of the dogs never had anti-rabies vaccination and vaccination status of 23 (31.1%) of the dogs was unknown. The percentage of vaccination among the attacking dogs in the urban areas (53.3%) was significantly higher than 13.8% observed in the rural areas (*P* < 0.003). There was a history of previous bite in four (5.4%) of the dogs, and aggressive behavior in three (4.1%) of the dogs. Sixteen (21.6%) of the dog were lactating and the peak month incidence of bite injury by lactating dog was July. The incidence of provocation before attack was higher among lactating dogs (20%) than nonlactating dogs (18.6%) (*P* = 0.905). Four (5.4%) of the dogs were killed soon after they attacked the victims. Two of the dogs died a few days after attacking the victims and cause of death was not determined. Fifty-eight (78.4%) of the dogs were alive and well beyond 10 days after bite whereas the fate of 10 (13.5%) of the dogs after the bites was unknown.

Majority of the victims, 39 (52.7%) presented within the first 6 h of injury whereas 19 (25.7%) and 16 (21.6%) presented in the hospital 7–24 h and >24 h after injury, respectively. Twenty (27%) of the patients received prehospital care before presentation to the hospital whereas 73% had no prehospital care. Sixty-four (86.5%) of the patients received a recommended doses of postexposure anti-rabies prophylaxis. All the victims received antibiotics and anti-tetanus toxoid. The mean and median length of hospital stay was 1.46 ± 0.32 days and 1 day, respectively.

In Table 3, majority of the victims 73 (98.7%) recovered fully whereas wound infection and clinical rabies were the two complications observed. The case of rabies was a 15-year-old male rural student who presented with clinical features of rabies 90 days after a bite in the leg by an abnormally aggressive stray dog of unknown vaccination status. He discharged self against medical advice after 3 days of hospitalization.

## Discussion

The incidence of dog bite-related injury treated in the hospital emergency room is within the worldwide range in published reports.^[15–17]^ The result of this study indicates a female gender bias in the sex distribution of victims of dog bite injuries. This is at variance with the preponderance of male victims reported in almost all published studies on dog bite-related injury.^[1,6,10,11,17–19]^ The reason for the predominance of female victims in this series is not evident.

In human-dog interactions, bilateral misinterpretation of signals and inappropriate reactions is the root cause of dog bite injury often times.^[21]^ Children who may not have the much-needed maturity to correctly interpret and react in rapidly changing interactions^[21]^ are at greater risk of injury. The risk of injury is even more among male children who are more aggressive in behavior and more likely to have dogs as pet compared to girls.^[22]^ Thus, in Figure 1, the male outnumber the female victims of dog bite in the first 14 years of life. However, in the setting of this study, most females (especially in adolescence and early young adulthood) are so scared at the sight of dogs and are more likely to exhibit inappropriate reaction, such as screaming and running that incite predatory behavior in dogs. These features perhaps explain the preponderance of females over males among the adolescents and young adult victims, and the higher incidence of attack among the females on the road/streets observed.

In this series, the preponderance of buttocks injury in the first 4 years of childhood correlated with buttocks as a common sites of dog bite in children reported by Ojuawo and Abdulkareem in Northern Nigeria^[23]^ but differs from the predominance of head and face injury in younger children in published reports from developed countries.^[10,13,22,24–27]^ In developed countries where most dogs are domestic pets and companion,^[28]^ while playing with familiar dogs, the licks on the face and head region of a child can result in a bite in the same anatomical site whenever there is bilateral misinterpretation of signals and inappropriate reaction. Meints *et al*. demonstrated that children especially the younger ones have intrusive inspection behavior that predisposes them to facial dog bite injury.^[29]^ This is an additional risk factor for bite injuries in the head region especially in a setting where most dogs are domestic pets, and perhaps further explains the preponderance of head and facial injury in children in developed countries. On the contrary, in developing countries, most dogs are free-loaming^[7,9]^ that are more likely to pursue and bite a victim (especially children) in buttocks and lower extremity.^[23]^ Furthermore, in the setting of this study, disposal of fecal waste and licking of the anal region of children to clean it after defecation is additional domestic function of dogs. However, the extent of involvement of this peculiar practice as a factor in the preponderance of buttocks injury in the first 4 years of childhood observed is not evident and requires further study.

The preponderance of extremity injury in this study is similar to the findings in other published reports on dog bite injury in general population.^[7,15,18–20,22]^ This pattern of anatomical distribution of injuries has been attributed to the use of hand and leg to ward off attacking dog, height of dog in relation to the victim, and the extremities being a better surface for biting than the trunk.^[22]^

**Figure 4:**
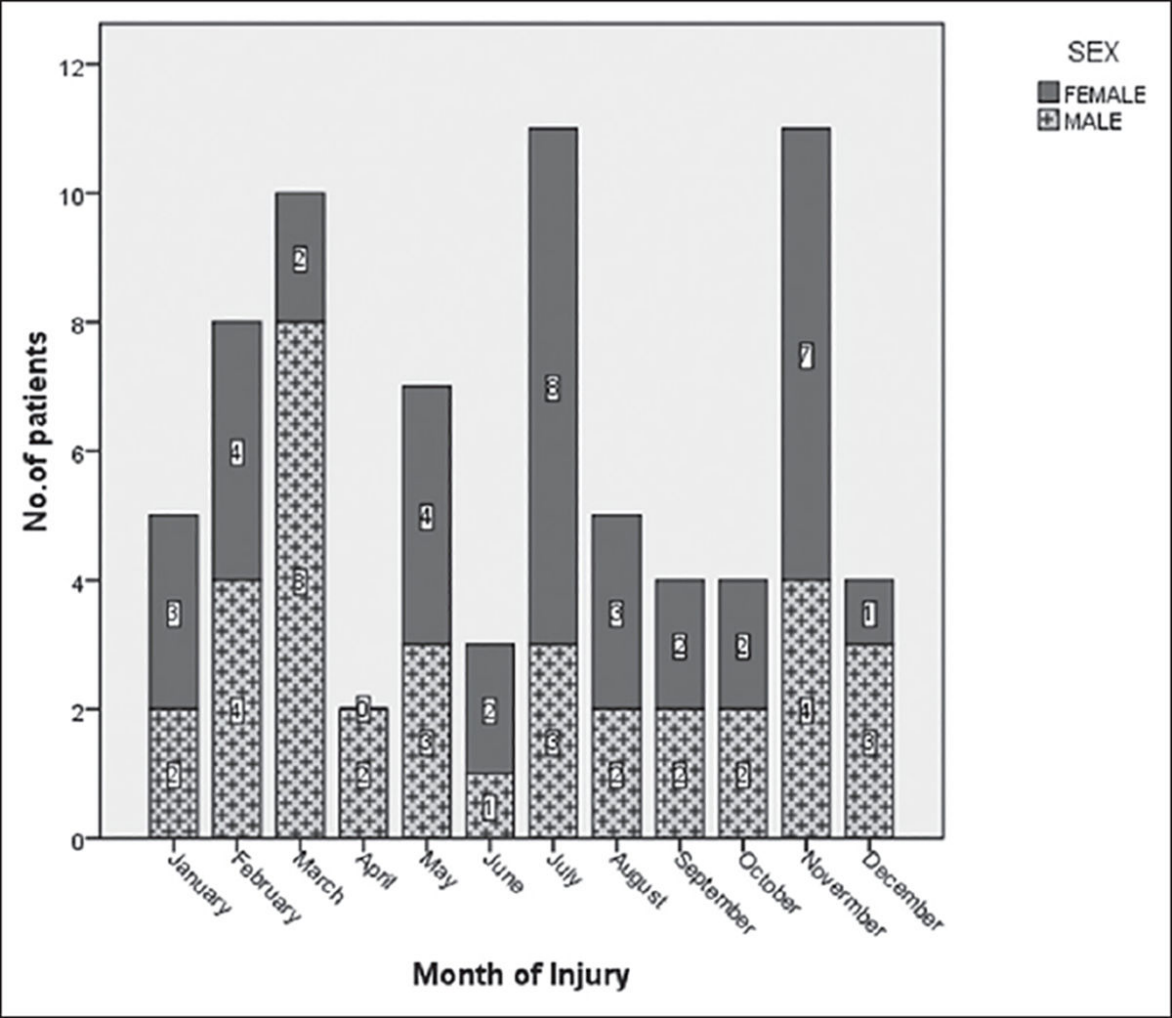
Incidence of dog bite injuries by gender and month of injury

In previous published reports, the preponderance of dog bite injury in summer was consistent with increased outdoor recreational activities and human-dog contact associated with the warm season in temperate zone countries.^[16,18,21,24]^ In the setting of this study, most of the children and youths play in compounds, and outing to playgrounds occur during the dry season. This perhaps explains the preponderance of dog bite injury in dry season observed. The reason/s for the variation in the peak month of incidence by gender as shown in Figure 4 is not evident and calls for a further study.

The incidence of unprovoked dog bite in this study is similar to the finding reported by Tenzin *et al*.^[15]^ in Bhutan but differs significantly from 35% reported by Parrish *et al*. in Pittsburgh.^[22]^ A recent publication on dog’s attacking behavior indicates that an unexpected dog bites rarely occur for no reason and unless a dog is sick all bites are provoked by something such as fear, excitement, surprises, and protection related needs.^[30]^ Thus, circumstances of attack perceived as unprovoked are actually due to reasons unrecognized by the victims. Hence, the very high incidence of unprovoked bite observed in this series suggests under-recognition of these reasons and inappropriate response among majority of the victims. This calls for the educational program to enlighten the public on the behavior of dogs and appropriate responses to prevent an unexpected bite.

The peak period incidence (6–11.59 am) of dog bite observed in this study differs from 6 to 11.59 pm reported by Parrish *et al*.^[22]^ This peak period of incidence and the predominance of dog bite injury in daytime is a reflection of prevailing pattern of lifestyle. In the setting of this study, most occupational and recreational activities, friend, and relative visitations occur in daytime whereas nightlife activity is relatively low. All these activities increase the likelihood of human-dog contact and perhaps explain the preponderance of bites in the daytime.

The proportion of patients that presented within the first 24 h is similar to the finding reported by Abubakar and Bakari in Northern Nigeria.^[17]^ In this series, the percentage of victims that presented to the emergency room without prehospital care is not too far from 86.4% reported by Ehimiyein *et al*.^[7]^ Thus, simple first aid measures (such as washing the wound with soap and copious amount of water) that reduce the risk of transmission of rabies was lacking in a significant proportion of the victims before presentation. Therefore, in our environment, emphasis on prehospital care is important in any educational program to enlighten the public on dog bite injury. The percentage of the victims that received postexposure anti-rabies vaccine is also similar to the finding reported by Abubakar and Bakari.^[17]^

The percentage of vaccinated and unvaccinated dogs observed is within the range reported in other developing countries.^[7,14,17,23]^ However, the percentage of dogs vaccinated in this series, especially among dogs in rural areas is too far below the 70% required to prevent the outbreak of dog rabies.^[31]^ This implies that the risk of transmission of rabies through dog bite is very high in our environment, and calls for a policy response to ensure higher coverage of anti-rabies vaccination of dogs.

The incidence of clinical rabies observed in this study is within the range in other published reports.^[14,17]^ However, timely presentation and intervention in the hospital after a bite by an abnormally aggressive stray dog could have prevented the clinical rabies observed.

The limitations of this study include its being a hospital and single center based one. The data obtained may not be a representation of the entire population.

## Conclusion

The incidence of dog bite injury treated in our hospital emergency room is within the worldwide range though the female gender bias in the sex distribution of the victims is unprecedented. There is variation in distribution of sex and anatomical site of injury by the age of victims. Majority of the attacks occurred in the compound and during dry season and were unprovoked. The percentage of anti-rabies vaccination among the dogs involved was very low, and majority of the patient did not receive prehospital care. These call for preventive strategies based on the observed pattern and a policy response to ensure higher coverage of anti-rabies vaccination.

## Figures and Tables

**Table 1:** Injury severity grade and circumstances of dog bite by ownership status of dog

	Ownership status of dog			χ^2^	*P*
	Owned	Stray	Total (%)		
Injury severity grade					
I	6	1	7 (9.5)	1.62	0.45
II	33	18	51 (68.9)		
III	12	4	16 (21.6)		
Circumstances bite					
Provoked	10	4	14 (18.9)	0.05	0.82
Unprovoked	41	19	60 (81.1)		

**Table 2:** Time and location/scene of dog bite injuries by ownership status of dog

	Ownership status of dog			χ^2^	*P*
	Owned	Stray	Total (%)		
Time of injury					
12:00 am-5:59 am	0	1	1 (1.4)	2.27	0.52
6:00 am-11.59 am	23	10	33 (44.6)		
12:00 pm-5.59 pm	18	8	26 (35.1)		
6:00 pm-11:59 pm	10	4	14 (18.9)		
Location					
Rural	17	12	29 (39.2)	2.36	0.12
Urban	34	11	45 (60.8)		
Place of occurrence/scene					
Home	13	0	13 (17.6)	13.33	0.01
Compound	26	9	35 (47.3)		
Street	3	2	5 (6.8)		
Road/highway	8	10	18 (24.3)		
Business premises/office	1	2	3 (4.1)		

**Table 3:** Outcome and complications of dog bite injuries (*n*=74)

Outcome	Number of patients (%)
Recovered	73 (98.7)
Sell-discharge against advice	1 (1.4)
Complications	
Rabies	1 (1.4)
Wound infection	1 (1.4)
